# KI-basierte Anwendungen in der medizinischen Bildverarbeitung

**DOI:** 10.1007/s00103-025-04093-7

**Published:** 2025-07-02

**Authors:** Timo Kepp, Hristina Uzunova, Jan Ehrhardt, Heinz Handels

**Affiliations:** 1 DFKI Forschungsbereich KI in der medizinischen Bild- und Signalverarbeitung, Deutsches Forschungszentrum für Künstliche Intelligenz, Ratzeburger Allee 160, 23562 Lübeck, Deutschland; 2 Institut für Medizinische Informatik, Universität zu Lübeck, Lübeck, Deutschland

**Keywords:** Deep Learning, Faltungsnetzwerke, Bildsegmentierung, Bildregistrierung, Generative KI, Deep learning, Convolutional neural networks, Image segmentation, Image registration, Generative AI

## Abstract

Die Verarbeitung medizinischer Bilder hat in der modernen Diagnostik und Therapie eine hohe Bedeutung. Eine automatisierte Verarbeitung und Analyse medizinischer Bilder hat das Potenzial, klinische Prozesse effizient und ressourcensparend zu beschleunigen. Sie eröffnet neue Möglichkeiten für eine verbesserte Patientenversorgung, ist jedoch aufgrund der hohen Variabilität, Komplexität und variierenden Qualität medizinischer Bilddaten sehr herausfordernd. In der medizinischen Bildverarbeitung wurden in den letzten Jahren die größten Fortschritte mit künstlicher Intelligenz (KI) erzielt, insbesondere durch tiefe neuronale Netze mithilfe von Deep Learning. Diese Verfahren werden erfolgreich zur Analyse medizinischer Bilddaten eingesetzt, etwa für Segmentierung, Registrierung und Bildsynthese.

Mithilfe KI-basierter Segmentierungsverfahren wird eine präzise Abgrenzung von Organen, Geweben oder pathologischen Veränderungen in hoher Qualität ermöglicht. Durch die Anwendung KI-basierter Bildregistrierungsverfahren können 3D-Planungsmodelle für komplexe Operationen beschleunigt erstellt werden, bei denen relevante Strukturen aus unterschiedlichen Bildern (CT, MRT, PET u. a.) oder Zeitpunkten aufeinander abgebildet werden. Mit generativen KI-Methoden können zusätzliche Bilddaten für das verbesserte Training von KI-Modellen erzeugt und so die Anwendungsmöglichkeiten von Deep Learning in der Medizin erweitert werden. Beispielhaft werden KI-Anwendungen in Radiologie, Ophthalmologie, Dermatologie und Chirurgie beschrieben und der praktische Nutzen und das Potenzial von KI-Anwendungen in der bildgestützten Diagnostik und Therapie aufgezeigt.

## Abstract (en)

The processing of medical images plays a central role in modern diagnostics and therapy. Automated processing and analysis of medical images can efficiently accelerate clinical workflows and open new opportunities for improved patient care. However, the high variability, complexity, and varying quality of medical image data pose significant challenges. In recent years, the greatest progress in medical image analysis has been achieved through artificial intelligence (AI), particularly by using deep neural networks in the context of deep learning. These methods are successfully applied in medical image analysis, including segmentation, registration, and image synthesis.

AI-based segmentation allows for the precise delineation of organs, tissues, or pathological changes. The application of AI-based image registration supports the accelerated creation of 3D planning models for complex surgeries by aligning relevant anatomical structures from different imaging modalities (e.g., CT, MRI, and PET) or time points. Generative AI methods can be used to generate additional image data for the improved training of AI models, thereby expanding the potential applications of deep learning methods in medicine. Examples from radiology, ophthalmology, dermatology, and surgery are described to illustrate their practical relevance and the potential of AI in image-based diagnostics and therapy.

## Einleitung

Medizinischen Bildern kommt in der modernen medizinischen Diagnostik und Therapie eine zentrale Rolle zu. Herausforderungen ergeben sich insbesondere aus der großen Variabilität der Bildinhalte, die sich aus der Vielzahl möglicher Krankheitsmuster und deren zeitlichen Veränderungen ergibt, sowie der Komplexität räumlicher oder zeitlicher Bilddaten. In den vergangenen Jahren wurden durch moderne Methoden der künstlichen Intelligenz (KI), insbesondere durch den Einsatz von Deep-Learning-Methoden, erhebliche Fortschritte erzielt.

Schlüsselprobleme der medizinischen Bildverarbeitung sind die Segmentierung anatomischer und pathologischer Strukturen wie Organe, Gewebe oder Tumoren, die Registrierung von Bilddaten sowie die Generierung synthetischer Trainingsdaten. Durch die Einführung tiefer neuronaler Netze (Deep Neural Networks, DNNs), die den Kern heutiger Deep-Learning-Methoden bilden, konnten in den letzten Jahren entscheidende Fortschritte erzielt werden, die die Diagnostik und Therapieplanung in neuer Qualität unterstützen.

Neben Segmentierung und Klassifikation umfasst der Einsatz KI-gestützter Verfahren heute auch Bildregistrierung und die Generierung synthetischer Trainingsdaten. So können korrespondierende Bildstrukturen (Organe, Tumoren etc.) in Aufnahmen unterschiedlicher Modalitäten wie Computer- und Magnetresonanztomografie (CT, MRT) räumlich aufeinander abgebildet werden. Ergänzend ermöglichen generative Deep-Learning-Modelle die Erstellung synthetischer, annotierter Bilddaten, die gezielt zur Erweiterung begrenzter Trainingsdatensätze eingesetzt werden können. Im weiteren Verlauf werden diese Ansätze und die zugrunde liegenden Modelle vertieft betrachtet.

In diesem Beitrag wird zunächst eine kurze Einführung in die grundlegenden Konzepte und Prinzipien von Deep-Learning-Methoden und -Netzwerken gegeben. Außerdem werden 3 zentrale methodische Forschungs- und Anwendungsbereiche der KI in der medizinischen Bildverarbeitung beleuchtet: die Bildsegmentierung, die Bildregistrierung und die Generierung synthetischer medizinischer Bilddaten. Die Netzwerkarchitekturen der eingesetzten DNNs sowie ihre grundsätzlichen Anwendungsmöglichkeiten werden vorgestellt. Danach werden beispielhafte KI-Anwendungen in der Radiologie, Ophthalmologie, Dermatologie und Chirurgie beschrieben und deren praktischer Nutzen sowie Potenzial für die medizinische Diagnostik und Therapie aufgezeigt. Ein Fazit schließt den Beitrag ab.

## KI in der medizinischen Bildverarbeitung

In der medizinischen Bildverarbeitung werden aufgrund der großen Erfolge vorrangig Deep-Learning-Methoden entwickelt, die auf DNNs in unterschiedlichen Architekturen basieren. Deep-Learning-Methoden gehören zu den maschinellen Lernverfahren, die sich von wissens-, regel- oder logikbasierten KI-Ansätzen dadurch unterscheiden, dass die in der medizinischen Anwendung oftmals komplexen Zusammenhänge nicht explizit beschrieben und repräsentiert werden müssen, sondern aus umfangreichen Daten im Rahmen eines Trainingsprozesses implizit erlernt werden. Sie unterscheiden sich damit von wissensbasierten Ansätzen, die auf explizit repräsentiertem Expertenwissen basieren und daraus Schlussfolgerungsketten ableiten. DNNs sind eine Klasse von Netzwerkarchitekturen, die im Deep Learning zum Einsatz kommen. In einem Trainingsprozess können sie anhand von Beispielen auch unbekannte Zusammenhänge zwischen Merkmalen und ihrer Bedeutung erlernen. Aufgrund der überragenden Erfolge dominieren DNNs die gesamte Forschung und Entwicklung im Bereich der medizinischen Bildverarbeitung so deutlich, dass mit dem Begriff *KI* in der Regel neuronale Netze gemeint sind.

### Grundlagen

**Abb. 1:**
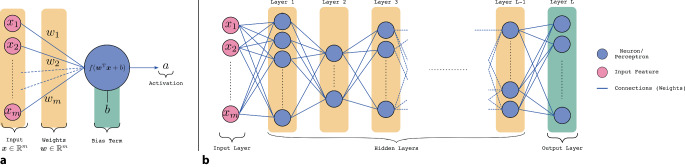
Illustration eines Neurons/Perzeptrons (**a**): Jede Komponente $x_{i}\in \left\{1,\ldots ,m\right\}$ des Eingangssignals wird mit einem Gewicht *w*_*i*_ gewichtet und aufsummiert. Zu der gewichteten Summe wird der Biasterm *b* hinzugefügt und zur Berechnung der Aktivierung *a* die nichtlineare Aktivierungsfunktion $f\left(\cdot \right)$ angewendet. Ein Multi-Layer-Perzeptron (**b**) besteht aus mehreren Schichten verbundener Neuronen und ist ein Beispiel für ein tiefes neuronales Netz (DNN)

Tiefe neuronale Netzwerke (DNNs) sind eine Klasse künstlicher neuronaler Netzwerke, die aus mehreren aufeinanderfolgenden Verarbeitungsschichten bestehen. Eine grundlegende Ausprägung stellt das sogenannte Multi-Layer-Perzeptron (MLP) dar, das aus mehreren Hierarchieebenen bzw. Schichten von Neuronen besteht [1]. Mehrere Neuronen auf einer Hierarchieebene bilden dabei eine einzelne Schicht (Layer) des Netzwerks (Abb. 1) und der Ausdruck *tief* wird dann verwendet, wenn viele solcher Schichten in dem Netzwerk auftreten. Obwohl jedes Neuron nur eine einfache Berechnung durchführt (eine gewichtete Summation der Eingangssignale und die Anwendung einer nichtlinearen Aktivierungsfunktion), kann ein DNN komplexe Berechnungsmodelle abbilden, indem die Netzwerkgewichte problemspezifisch erlernt werden. Die als Lernstrategie verwendete *Backpropagation* (rückwärtsgerichtete Fehlerweitergabe; [2]) benötigt dafür eine differenzierbare Zielfunktion und einen (umfangreichen) Trainingsdatensatz.

Ein Nachteil von MLPs in der medizinischen Bildverarbeitung ist, dass die Anzahl der zu lernenden Parameter quadratisch mit der Eingabegröße ansteigt. Bei medizinischen Bilddaten mit Millionen von Pixeln oder Voxeln ist eine direkte Nutzung der Rohdaten nicht praktikabel, weshalb zunächst vorgegebene Merkmale (sog. Handcrafted Features) extrahiert wurden.

**Abb. 2:**
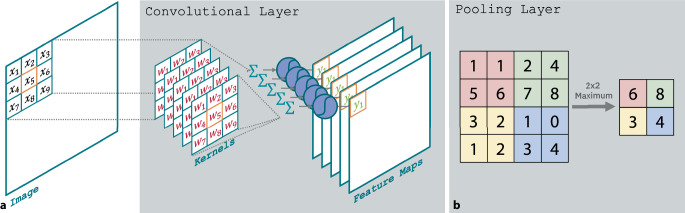
Funktionsweise der Faltungsschicht und Pooling-Schicht. Bei Convolutional Neural Networks (CNNs) werden Kernelgewichte zu Netzwerkparametern. Mehrere Kernels bewegen sich über das Bild/Eingangssignal und generieren Merkmalskarten (**a**). Pooling-Schichten reduzieren deren Größe, indem benachbarte Merkmale zusammengefasst werden (**b**)

Ein entscheidender Fortschritt war die Entwicklung von Faltungsschichten [3], bei denen jedes Neuron nur eine lokale Nachbarschaft betrachtet (Abb. 2a). Das bedeutet, dass die gewichtete Summation und Anwendung der Aktivierungsfunktion nur für räumlich benachbarte Merkmale erfolgen. Dies reduziert die Anzahl der Parameter und den Speicherbedarf erheblich. Ergänzend dazu gibt es Pooling-Schichten, die die Eingabedaten durch Mittelwertbildung oder Maximumoperationen auf weniger Werte reduzieren (Abb. 2b). Zusammen mit Aktivierungsfunktionen und weiteren spezialisierten Schichten bilden Faltungs- und Pooling-Schichten die Grundlage moderner *Faltungsnetzwerke* (Convolutional Neural Networks, CNNs).

Auch Netzwerkarchitekturen, die ursprünglich für die Sprachverarbeitung entwickelt wurden, finden in der medizinischen Bildverarbeitung Anwendung. So werden *Recurrent Neural Networks* (RNNs) und *Long Short-Term Memory* (LSTM) *Networks* in der Bildverarbeitung eingesetzt, um sequenzielle oder zeitliche Abhängigkeiten zu modellieren, z. B. bei zeitlichen Bilddaten oder Bildsequenzen. In jüngerer Zeit kommen verstärkt transformerbasierte Architekturen zum Einsatz, insbesondere *Vision Transformer* (ViT) und vergleichbare Modelle [4, 5]. Wesentlicher Bestandteil dieser Modelle sind Aufmerksamkeitsschichten (Attention Layers), deren Grundidee darin besteht, dass ein Modell selektiv auf relevante Teile der Eingabe fokussiert, anstatt alle Informationen gleich zu gewichten [6]. Dadurch können Transformer-Modelle komplexe und umfangreiche Daten, wie z. B. medizinische Bilder, effizienter verarbeiten. Im Gegensatz zu CNNs, die lokal begrenzt sind, werden in Transformer-Modellen Beziehungen zwischen beliebigen Regionen eines Bildes modelliert. Allerdings erhöht sich dadurch die Komplexität, wodurch transformerbasierte Modelle oft größere Datensätze und zusätzliche Datenaugmentierung benötigen, um im Vergleich zu CNNs konkurrenzfähig zu sein. Aufgrund der begrenzten Verfügbarkeit geeigneter Trainingsdaten im medizinischen Kontext sind CNNs daher nach wie vor die vorherrschende Netzwerkarchitektur in der medizinischen Bildverarbeitung.

### Einordnung von KI-Methoden in der medizinischen Bildverarbeitung

Eine mögliche Taxonomie zur Einordnung von KI-Methoden, wie beispielsweise Deep-Learning-Modellen auf der Basis von DNNs, in der medizinischen Bildverarbeitung orientiert sich an 3 Achsen: der Mustererkennungsaufgabe, der anatomischen Körperregion und der bildgebenden Modalität.

Typische Mustererkennungsaufgaben in der medizinischen Bildverarbeitung sind die Klassifikation oder Detektion von Krankheiten oder Pathologien, die Segmentierung von anatomischen oder pathologischen Strukturen und die Registrierung bzw. Bildfusion unterschiedlicher Bilddaten. Diese Mustererkennungsaufgaben werden abhängig vom betrachteten Krankheitsbild für unterschiedliche anatomische Regionen und verschiedene Bildmodalitäten erlernt. Bildgebende Modalitäten wie Röntgen, MRT oder CT liefern dabei einzigartige Informationen über die anatomischen Strukturen oder physiologischen Prozesse, die in der ausgewählten Körperregion untersucht werden sollen. Zum Training eines DNN wird ein umfangreicher Datensatz annotierter medizinischer Bilddaten benötigt, der mit der gewählten Modalität, Körperregion und medizinischen Fragestellung korrespondiert. Dieser Datensatz ist für das DNN von entscheidender Bedeutung, um Muster und Merkmale zu lernen, die für die gewünschte Aufgabenstellung relevant sind. Die konkrete Netzwerkarchitektur des DNN wird durch die zugrunde liegende Mustererkennungsaufgabe bestimmt und an die spezifischen Bildeigenschaften angepasst.

### KI-Modelle für die Bildsegmentierung

**Abb. 3:**
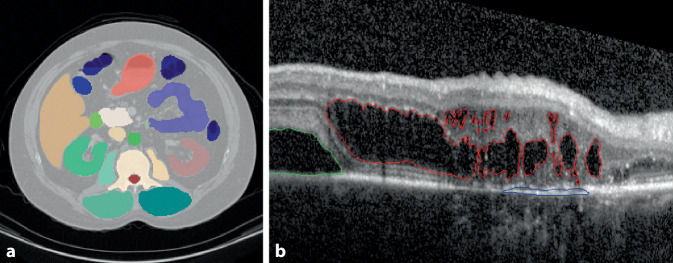
Segmentierungsbeispiele für verschiedene medizinische Anwendungsbereiche. **a** Segmentierung verschiedener Organe im Computertomografie-(CT-)Bild [48]. **b** Segmentierung pathologischer Strukturen in der optischen Kohärenztomografie (OCT) der Retina

Die Segmentierung ist ein zentrales Verfahren zur Erkennung und Vermessung von Objekten in Bildern. Ihr Ziel ist die Unterteilung des Bildes in inhaltlich zusammenhängende Bereiche, sogenannte Segmente. In medizinischen Bilddaten liefert die Segmentierung wertvolle Informationen über die Form, Größe und Lage anatomischer sowie pathologischer Strukturen (Abb. 3).

Die Erstellung einer manuellen Segmentierung ist zeitaufwendig und kann oftmals nur von medizinischen Experten durchgeführt werden. Dabei stellen stark schwankende Bildqualität, Rauschen und Artefakte eine große Herausforderung für die Segmentierung dar. CNNs ermöglichen eine automatische pixelgenaue Segmentierung mit höherer Genauigkeit, Robustheit und Anpassungsfähigkeit als klassische Verfahren wie Schwellenwertmethoden, kanten- oder regionenbasierte Ansätze oder modellbasierte Verfahren. Kennzeichnend ist dabei ihre Fähigkeit zur automatischen Merkmalsextraktion. Dabei wird jeder zu segmentierende Bildpunkt durch einen umgebenden Bildausschnitt (Patch) repräsentiert, der als Eingabe für das CNN dient [7]. Dagegen sind *Fully Convolutional Networks* (FCN; [8]) darauf ausgelegt, ganze Bilder direkt zu segmentieren. Durch den Verzicht auf vollständig verbundene Schichten arbeiten sie effizienter und unabhängig von der Bildgröße. Für die medizinische Anwendung ist insbesondere das *U‑Net* [9] von herausragender Bedeutung.

**Abb. 4:**
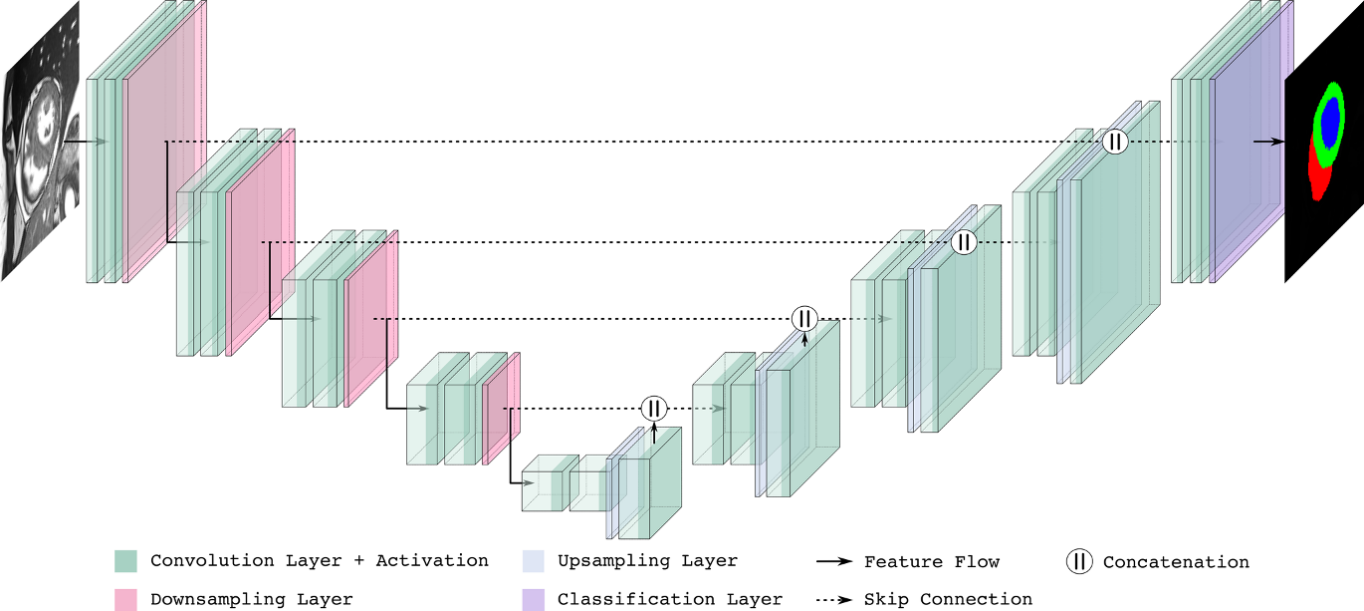
U‑Net-Architektur zur Segmentierung der Herzventrikel in Magnetresonanztomografie-(*MRT-*)Bilddaten

Das U‑Net-Modell besitzt eine symmetrische Encoder-Decoder-Architektur, welche die namensgebende U‑Form bildet. Über sogenannte Skip Connections, also direkte Verbindungen zwischen Schichten gleicher Auflösung, werden hoch- und niedrigauflösende Merkmale und somit globale und lokale Objekteigenschaften kombiniert erlernt. Dies ermöglicht präzise Segmentierungen, selbst bei kleinen Trainingsdatensätzen. In Abb. 4 ist die U‑Net-Architektur schematisch dargestellt. Mittlerweile hat sich das U‑Net als Benchmark für verschiedene medizinische Bildsegmentierungsaufgaben [10] durchgesetzt und hat zahlreiche Weiterentwicklungen [11, 12] inspiriert. So wird in [13] das *no-new-U-Net* (nnU-Net) vorgestellt, das als selbst konfigurierendes U‑Net alle relevanten Hyperparameter zur automatischen Anpassung an neue Datensätze automatisch ermittelt. Das nnU-Net wird inzwischen für eine große Bandbreite verschiedener medizinischer Segmentierungsaufgaben eingesetzt und dient unter anderem als Grundlage für Anwendungen wie den *TotalSegmentator* [14], ein vortrainiertes nnU-Net zur Segmentierung anatomischer Strukturen in CT- und MRT-Bilddaten.

### KI-Modelle für die Bildregistrierung

Bildregistrierung ist der Prozess, bei dem mehrere Bilder eines Objekts mittels einer Transformation (z. B. Translation, Skalierung, Rotation oder auch komplexere Deformationen) räumlich zueinander ausgerichtet werden, sodass korrespondierende Bildstrukturen aufeinander abgebildet werden [15–18]. In der medizinischen Bildverarbeitung wird dies genutzt, um Bilder aus unterschiedlichen Modalitäten (z. B. CT und MRT) oder Zeitpunkten (z. B. vor und nach einer Behandlung) zu vergleichen und zu kombinieren. Anwendungen umfassen die Fusion diagnostischer Bilddaten, die Operationsplanung, das Monitoring von Tumorwachstumsprozessen und die Überwachung von Krankheitsfortschritten. Klassische Verfahren zur Bildregistrierung nutzen häufig rechenaufwendige iterative Optimierungsalgorithmen, um die gesuchten Transformationsparameter zu bestimmen. Das Optimierungskriterium ist dabei ein Distanz- bzw. Ähnlichkeitsmaß, das die räumliche Übereinstimmung der transformierten Bilder misst. Registrierungsalgorithmen können in lineare und nichtlineare Verfahren eingeteilt werden. Lineare Transformationen, wie rigide oder affine Modelle, werden z. B. zur groben Fusion verschiedener Modalitäten (z. B. CT- und MRT-Daten einer Person) eingesetzt, während nichtlineare Transformationen komplexe lokale Deformationen ermöglichen und z. B. bei der Analyse von Organbewegungen [19, 20] oder bei populationsbasierten Analysen morphologischer Unterschiede und Gemeinsamkeiten in einem Patientenkollektiv [21] Anwendung finden.

Da die langen Laufzeiten iterativer Registrierungsverfahren für Echtzeitanwendungen ungeeignet sind, werden neuronale Netze genutzt, um die Transformation direkt aus den Eingabebildern vorherzusagen. Für *überwachte lernbasierte Registrierungsverfahren* werden die benötigten Grundwahrheiten (Transformationsparameter oder Deformationen) entweder mittels klassischer Registrierungsverfahren bestimmt [22, 23] oder es werden generative Modelle genutzt, um geeignete Trainingsbilder mit zugehörigen Transformationen zu erzeugen [24, 25].

Von entscheidender Bedeutung für *unüberwachte lernbasierte Registrierungsverfahren* war die Einführung des „Spatial Transformation Layer“ [26], eines differenzierbaren Moduls, das die räumliche Manipulation von Bilddaten und Merkmalen in CNNs erlaubt und das Lernen der Transformationsparameter durch Backpropagation ermöglicht. Basierend auf diesem Modul wurden Registrierungsnetzwerke entwickelt, die unter Nutzung der Optimierungskriterien klassischer Registrierungsverfahren unüberwacht trainiert werden [27, 28].

Eine wesentliche Herausforderung lernbasierter Registrierungsverfahren ist die Robustheit bzgl. großer Deformationen, d. h., wenn korrespondierende Strukturen in den zu registrierenden Bildern weit voneinander entfernt sind. Neben der Integration von Multi-Resolution-Ansätzen in CNNs [29] erwies sich auch der globale Aufmerksamkeitsmechanismus von Transformer-Modellen als geeignet, um dieses Problem anzugehen [30]. Eine weitere aktuelle Forschungsrichtung ist die Entwicklung generalisierter Modelle, d. h. Netzwerke zu trainieren, die möglichst unabhängig von spezifischen Bildmodalitäten, Bildauflösungen, Patientenkollektiv und Körperregion sind. Hier erwiesen sich erste Ansätze, die auf synthetisierte Daten trainiert werden, als äußerst vielversprechend [31].

### Generative KI zur Erzeugung synthetischer medizinischer Bilder

Generative Modelle, wie z. B. ChatGPT von OpenAI zur Textgenerierung, haben in den letzten Jahren stark an Popularität gewonnen und haben sich auch als vielfältig einsetzbare Instrumente in der medizinischen Bildverarbeitung etabliert [32].

Hintergrund ist, dass die Qualität trainierter KI-Modelle oft stark von der Verfügbarkeit von annotierten Trainingsdaten abhängt. Bei medizinischen Bilddaten ist dies besonders problematisch, da deren Erhebung kosten- und zeitintensiv ist und oft durch Datenschutz- und Privatsphärenbedenken eine Nutzung eingeschränkt wird. Zusätzlich sind medizinische Daten häufig heterogen und enthalten Rauschen oder Artefakte, abhängig von den verwendeten Geräten. Seltene Krankheiten sind in den Daten oft unterrepräsentiert, was zu unausgewogenen Datensätzen führt.

**Abb. 5:**
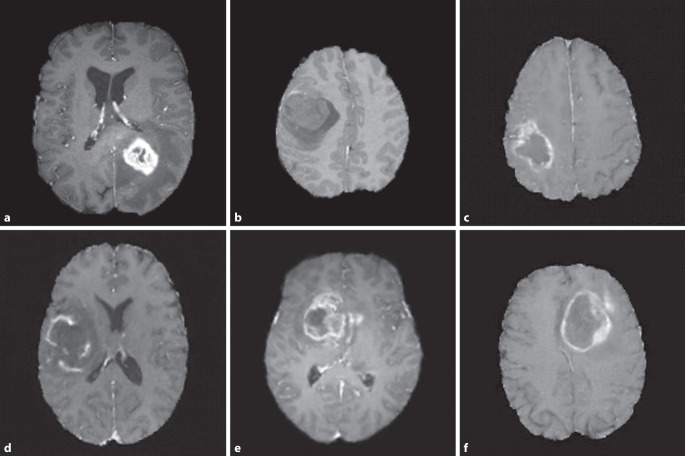
Reale und synthetische T1-gewichtete MRT-Bilder (Magnetresonanztomografie) des Gehirns mit Kontrastmittel. **a–c** Originalaufnahmen mit Glioblastomen aus dem BRATS-Datensatz (Brain Tumor Segmentation; [51]). **d–f** Künstlich erzeugte Bilder aus einem Diffusionsmodell. Hinweis: Alle Bilder sind „skullstripped“ – der Schädel wurde aus Datenschutzgründen entfernt

Hier schafft die Synthese medizinischer Bilddaten mittels generativer KI neue Möglichkeiten, da hierdurch eine unbegrenzte Anzahl von Bildern generiert werden kann, die zudem keine direkten Patienteninformationen enthalten und somit keinen Datenschutzbeschränkungen unterliegen (Beispiele in Abb. 5). Die generierten Daten können gezielt nach bestimmten Kriterien erstellt werden, z. B. artefaktfreie Bilder oder Bilder mit seltenen Erkrankungen, was zu ausgewogeneren Trainingsdatensätzen führt. Außerdem erzeugen viele generative Modelle automatisch gelabelte oder annotierte Bilddaten, die für das Training neuronaler Netze verwendet werden können. Im Allgemeinen kann angenommen werden, dass generative Modelle die Verteilung echter Daten abbilden können. Auch wenn kleine Unterschiede zwischen generierten und echten Daten zu erkennen sind, haben unzählige Experimente bereits gezeigt, dass synthetische Bilder in großen repräsentativen Datensätzen für das Training neuronaler Netze gut geeignet sind.

**Abb. 6:**
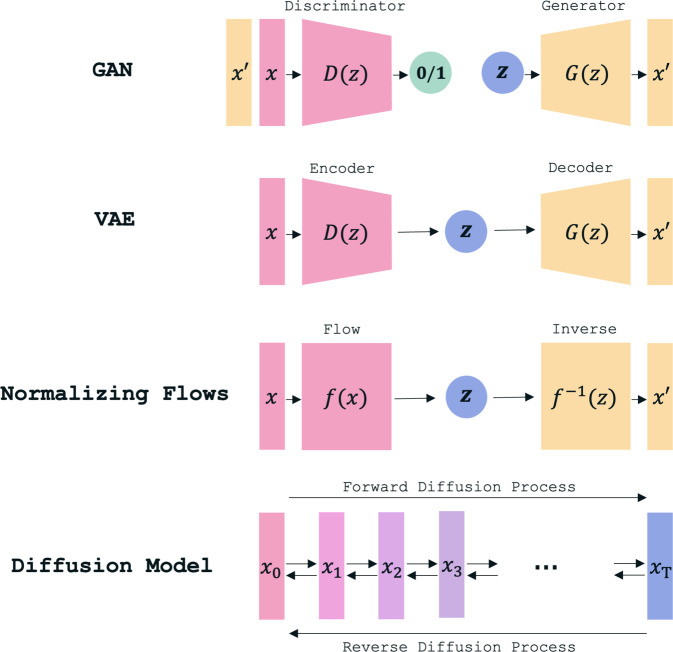
Bespiele für generative Modelle. Von oben nach unten: Generative Adversarial Network (GAN), Variational Autoencoder (VAE), Normalizing Flows und Diffusion Model

Häufig eingesetzte generative Modelle (Abb. 6) sind unter anderem *Autoencoder* (AEs) und *Variational Autoencoder* (VAEs). Das sind neuronale Netze mit 2 Teilen: einem Encoder, der Eingaben wie Bilder in eine kompakte codierte Darstellung umwandelt, und einem Decoder, der aus dem Code das ursprüngliche Bild rekonstruiert. VAEs erweitern dieses Prinzip, indem sie für die Generierung neuer Bilder geeignet sind. Zwar sind die generierten Bilder hier häufig verschwommen und weisen eine reduzierte Auflösung auf, trotzdem eignen sich diese Modelle gut für die unüberwachte Detektion von Pathologien in medizinischen Daten [33, 34].

Um realistischere Daten zu synthetisieren, eignen sich Methoden wie *Generative Adversarial Networks* (GANs), *Normalizing Flows* und *Diffusion Models*. GANs bestehen aus einem Generator und einem Discriminator, die in einem *Katz-und-Maus-Spiel* lernen, sehr realistische Bilder zu erzeugen. Beide Netze verbessern sich gegenseitig: Der Generator lernt, realistische Bilder zu erzeugen, und der Discriminator, diese besser zu erkennen. Die so synthetisierten Bilder können verwendet werden, um weitere neuronale Netze zu trainieren und zu evaluieren, ohne einen signifikanten Präzisionsverlust zu erleiden [35–37]. Normalizing Flows modellieren komplexe Wahrscheinlichkeitsverteilungen, indem sie eine einfache Verteilung, wie eine Normalverteilung, schrittweise durch invertierbare Transformationen in eine Zielverteilung umwandeln. Diese Transformationen werden durch neuronale Netze realisiert, wodurch Daten generiert, analysiert und rekonstruiert werden können. Normalizing Flows finden Anwendung in Bereichen wie Bildgenerierung, Dichteschätzung und Anomaliedetektion. Diffusion Models nutzen stochastische Prozesse, um Daten zu generieren. Im Vorwärtsprozess wird Rauschen schrittweise zu realen Daten hinzugefügt, bis sie einer Gauß-Verteilung entsprechen. Der Rückwärtsprozess entfernt das Rauschen und rekonstruiert realistische Daten. Diffusion Models erzeugen besonders realistische, detailreiche Bilder und bieten vielseitige Konditionierungsmöglichkeiten.

## Medizinische Anwendungen von bildgestützten KI-Methoden

In der medizinischen Diagnostik werden KI-Methoden eingesetzt, um automatisch Krankheitsmuster in Bilddaten zu detektieren und zu erkennen. Die KI-basierte Detektion ist insbesondere bei der Analyse von medizinischen Bildern aus Screening-Untersuchungen von Bedeutung. Mithilfe von KI-basierter Segmentierung wird eine präzise Abgrenzung von Organen, Geweben oder pathologischen Veränderungen in hoher Qualität ermöglicht. Die Segmentierung bildet die Grundlage für eine stärker quantitativ ausgerichtete Radiologie, bei der automatisch quantitative Kenngrößen wie Flächen, Volumina oder der maximale Durchmesser für die segmentierten Gewebe, Organe und Tumore, Läsionen etc. berechnet werden können. So ist z. B. das Tumorvolumen bei der Laufzeitkontrolle von Tumoren unter Therapie von Bedeutung, bei der die zwischen verschiedenen Zeitpunkten ermittelte Veränderung des Tumorvolumens einen wichtigen Indikator zur Bewertung des Therapieerfolgs darstellt. Aber auch für die verbesserte bildgestützte Strahlentherapie, die Planung operativer Eingriffe und die bildgestützte Navigation der Chirurgin oder des Chirurgen im Operationssaal sind KI-basierte Segmentierungs- und Erkennungsverfahren bedeutsam. So müssen in der Strahlentherapie vor der Bestrahlung der zu bestrahlende Tumor und die umliegenden gesunden Organe segmentiert werden, um so einen optimierten Bestrahlungsplan zu generieren, sodass der Tumor bei der Bestrahlung maximal geschädigt und die umliegenden gesunden Organe geschont werden.

Durch die Anwendung KI-basierter Bildregistrierungsverfahren können 3D-Planungsmodelle für komplexe Operationen beschleunigt erstellt werden, bei denen die verschieden relevanten Organe, Pathologien, Gefäße etc. aus Bildern unterschiedlicher Modalitäten extrahiert und gemeinsam in einem 3D-Planungsszenario dargestellt werden. Auch Verlaufskontrollen oder quantitative Bewegungsanalysen des Herzens oder der atmungsbedingt bewegten inneren Organe können mit diesen Verfahren ermöglicht werden. Nachfolgend werden verschiedene Anwendungen von bildgestützten KI-Methoden in der Radiologie, Dermatologie, Augenheilkunde und der Chirurgie beispielhaft beschrieben.

### Radiologie

In der Radiologie ist eine Vielzahl von KI-Systemen bereits in der Erprobung oder auf dem Weg in die klinische Anwendung. Hierbei handelt es sich um auf dedizierte Fragestellungen optimierte KI-Systeme. Medizinische Anwendungsbereiche von KI-Systemen in der Radiologie sind etwa die Detektion von Brustkrebs in Mammografien oder von Schlaganfällen in 3D-CT- oder 3D-MRT-Bildfolgen. Beispielhaft sollen die Vorteile, die durch den Einsatz von KI-Systemen in der Praxis erzielt werden können, anhand der Erfolge der KI-basierten Analyse von Mammografien illustriert werden.

So wurden KI-Modelle eingesetzt und evaluiert, um Bilder einer der weltweit größten Mammografie-Studien PRAIM auszuwerten, bei der Daten von über 460.000 Frauen zwischen 2021 und 2023 an insgesamt 12 Standorten in Deutschland im Rahmen des Mammografie-Screening-Programms erhoben wurden [38]. Zur Untersuchung der Leistungsfähigkeit der eingesetzten KI-Modelle wurde ca. die Hälfte der Mammografien mithilfe von KI-Modellen ausgewertet, während die andere Hälfte durch Doppelbefundung von Radiologinnen und Radiologen untersucht wurde. Hierbei konnte durch den Einsatz automatischer KI-Detektionsalgorithmen die Entdeckungsrate für Brustkrebs um fast 18 % gesteigert werden. Positiv war darüber hinaus, dass es dabei nicht vermehrt zu falschem Alarm oder unnötigen Zusatzuntersuchungen durch den Einsatz der KI-Modelle gekommen ist. Die Analyse im Rahmen der von der Universität zu Lübeck und dem Universitätsklinikum Schleswig-Holstein (UKSH), Campus Lübeck, in Zusammenarbeit mit der Firma Vara durchgeführten Studie hat zudem gezeigt, dass der Einsatz der KI-Software die Arbeitslast von Radiologinnen und Radiologen ohne Qualitätsverlust reduzieren kann. Ein wichtiges Ergebnis war auch, dass die Brustkrebsentdeckungsrate um 16,7 % höher läge und die Anzahl der Wiedereinbestellungen um 15 % sinken würde, wenn alle von der automatischen KI-Analyse als unauffällig eingestuften Fälle nicht mehr durch Menschen befundet würden. Diese Ergebnisse machen exemplarisch das enorme Potenzial von KI-Modellen in der bildgestützten Medizin deutlich.

### Augenheilkunde und Dermatologie

Der Einsatz von KI ist in der Augenheilkunde und Dermatologie äußerst vielversprechend, da Diagnose, Therapieplanung und Verlaufskontrolle durch eine automatische Bilderkennung unterstützt werden können. Bereits 2016 wurde der Nutzen der KI in der Augenheilkunde gezeigt, indem ein Deep-Learning-Algorithmus entwickelt wurde, der diabetische Retinopathie in Fundusbildern mit der Genauigkeit einer erfahrenen Augenärztin bzw. eines erfahrenen Augenarztes erkennen konnte [39]. Inzwischen wird KI eingesetzt, um die Diagnose und Behandlung verschiedener Augenerkrankungen wie Glaukom oder altersbedingter Makuladegeneration (AMD) zu unterstützen. So dienen Algorithmen zur Segmentierung einzelner Retinaschichten bei der Glaukomdiagnostik, wohingegen die Erkennung und Segmentierung von krankhaften Flüssigkeitsansammlungen einen wichtigen AMD-Biomarker darstellen [40]. Ferner kann KI zur Prognoseschätzung von Krankheitsverläufen genutzt werden [41, 42], um ideale Behandlungszeitpunkte zu bestimmen, die insbesondere bei der AMD für den Erhalt der Sehkraft kritisch sind. Eine weitere vielversprechende Entwicklung ist die Einführung von Homecare-Lösungen [43], die Patientinnen und Patienten ermöglichen, ihren Krankheitszustand selbstständig und regelmäßig zuhause zu überwachen. In Kombination mit KI-basierten Analysemethoden [44] könnte dies eine kontinuierliche Überwachung von Augenerkrankungen ermöglichen und so die Früherkennung sowie das Management von Erkrankungen wie AMD oder diabetischer Retinopathie verbessern.

Auch in der Dermatologie haben KI-Anwendungen früh Einzug gehalten und werden vor allem zur Malignitätseinschätzung auf Dermatoskopiebildern eingesetzt. Hier konnte bereits demonstriert werden, dass die KI auf Expertenniveau oder besser Hautkrebs erkennen kann [45]. Weitere Einsatzbereiche liegen in der unterstützenden Analyse histopathologischer Schnitte, etwa bei der Resektionsplanung von Basalzellkarzinomen.

Bereits heute sind KI-Algorithmen in Kombination mit Medizinprodukten auf dem europäischen Markt zugelassen, wie der *Moleanalyzer Pro* der Firma FotoFinder GmbH, der durch ein KI-Scoring die Malignitätseinschätzung unterstützt [46]. Zudem werden KI-Anwendungen in Form von Smartphone-Anwendungen angeboten, um Patientinnen und Patienten auch außerhalb der Klinik ein mobiles Screening auf Hautkrebs zu ermöglichen.

### Chirurgie

Bildverarbeitende KI besitzt enormes Potenzial, Präzision, Sicherheit und Effizienz chirurgischer Eingriffe zu steigern. Zentrale Einsatzmöglichkeiten sind präoperative Planung, postoperative Analyse, intraoperative Unterstützung sowie Training und Simulation. Im Bereich der prä- und postoperativen Planung können KI-Algorithmen vor allem dort in der Bildanalyse eingesetzt werden, wo anatomische und pathologische Strukturen präzise identifiziert, quantifiziert und dreidimensional modelliert werden sollen [47, 48]. Durch KI-basierte Segmentierungs- und Registrierungsverfahren können 3D-Planungsmodelle für komplexe Operationen erstellt werden, in denen relevante Organe, Pathologien, Gefäße und weitere Strukturen aus unterschiedlichen Bildmodalitäten (CT, MRT, PET etc.) extrahiert und gemeinsam in einem 3D-Planungsszenario zur verbesserten Operationsplanung visualisiert werden.

Weiterhin können KI-basierte Registrierungsmethoden eingesetzt werden, um die präoperativ generierten Bilder der Patientin oder des Patienten gemeinsam mit den erstellten Planungsinformationen während der Operation maßstabsgetreu im Operationssaal zu visualisieren und die Chirurginnen und Chirurgen durch bildgestützte Navigation zu unterstützen. Die erstellten 3D-Patientenmodelle können zugleich auch zur Planung und Steuerung des Einsatzes von OP-Robotern genutzt werden [49].

Ein anderer Bereich der Anwendung von KI-Methoden in der Chirurgie sind das Training und die Unterstützung in der Ausbildung von chirurgischem Personal. Hierbei werden auf der Grundlage der 3D-Patientenmodelle realistische Virtual-Reality-Simulationen durchgeführt, sodass komplexe chirurgische Eingriffe virtuell geübt werden können [50].

## Fazit

Die Einführung von KI-Methoden, insbesondere DNNs, hat die medizinische Bildverarbeitung grundlegend verändert. Aktuelle Forschung untersucht etwa, wie KI-Modelle trotz kleiner Trainingsmengen und begrenzter Ressourcen in hoher Qualität trainiert werden können oder wie sich ihre Erklärbarkeit verbessern lässt. Die durch KI-Methoden erzielten Erfolge haben die Forschung im Bereich der medizinischen Bildverarbeitung nachhaltig geprägt. Verbesserungen in Qualität und Robustheit KI-basierter Analysen ermöglichen inzwischen in vielen Bereichen den praktischen Einsatz von KI-Systemen. Ein deutliches Zeichen für die klinische Relevanz ist, dass die US-amerikanische Food and Drug Administration (FDA), zuständig für die Zulassung von Medizinprodukten und Software im Gesundheitswesen, bereits rund 800 KI-Anwendungen für den klinischen Einsatz freigegeben hat. Intelligente KI-Systeme werden die zukünftige Entwicklung der Medizin maßgeblich prägen – nicht zuletzt aufgrund erwarteter Effizienzgewinne und Arbeitserleichterungen in der bildgestützten Diagnostik und Therapie.

## References

[1] LeCun Y, Bengio Y, Hinton G (2015) Deep learning. Nature 521:436–444. 10.1038/nature14539 26017442 10.1038/nature14539

[2] Rumelhart DE, Hinton GE, Williams RJ (1986) Learning representations by back-propagating errors. Nature 323:533–536. 10.1038/323533a0

[3] LeCun Y, Bengio Y (1998) Convolutional networks for images, speech, and time series. In: The handbook of brain theory and neural networks. MIT Press, Cambridge, S 255–258

[4] Dosovitskiy A, Beyer L, Kolesnikov A et al (2021) An image is worth 16x16 words: transformers for image recognition at scale, International Conference on Learning Representations, https://openreview.net/pdf?id=YicbFdNTTy

[5] Han K, Wang Y, Chen H et al (2023) A survey on vision transformer. IEEE Trans Pattern Anal Mach Intell 45:87–110. 10.1109/TPAMI.2022.3152247 35180075 10.1109/TPAMI.2022.3152247

[6] Vaswani A, Shazeer N, Parmar N et al (2017) Attention is all you need. In: Proceedings of the 31st International Conference on Neural Information Processing Systems. Curran Associates, Red Hook, S 6000–6010

[7] Pereira S, Pinto A, Alves V, Silva CA (2016) Brain tumor segmentation using convolutional neural networks in MRI images. IEEE Trans Med Imaging 35:1240–1251. 10.1109/TMI.2016.2538465 26960222 10.1109/TMI.2016.2538465

[8] Long J, Shelhamer E, Darrell T (2015) Fully convolutional networks for semantic segmentation. In: 2015 IEEE Conference on Computer Vision and Pattern Recognition (CVPR). IEEE, Boston, S 3431–3440 10.1109/TPAMI.2016.2572683 27244717

[9] Ronneberger O, Fischer P, Brox T (2015) U‑net: convolutional networks for biomedical image segmentation. In: Navab N, Hornegger J, Wells WM, Frangi AF (Hrsg) Medical Image Computing and Computer-Assisted Intervention—MICCAI 2015. Springer, Cham, S 234–241

[10] Kepp T, Droigk C, Casper M et al (2019) Segmentation of mouse skin layers in optical coherence tomography image data using deep convolutional neural networks. Biomed Opt Express 10:3484–3496. 10.1364/BOE.10.003484 31467791 10.1364/BOE.10.003484 PMC6706029

[11] Çiçek Ö, Abdulkadir A, Lienkamp SS et al (2016) 3D U‑net: learning dense volumetric segmentation from sparse annotation. In: Ourselin S, Joskowicz L, Sabuncu MR et al (Hrsg) Medical image computing and computer-assisted intervention—MICCAI 2016. Springer, Cham, S 424–432

[12] Milletari F, Navab N, Ahmadi S‑A (2016) V‑net: fully Convolutional neural networks for volumetric medical image segmentation. In: 2016 Fourth International Conference on 3D Vision (3DV). IEEE, Stanford, S 565–571

[13] Isensee F, Jaeger PF, Kohl SAA et al (2021) nnU-Net: a self-configuring method for deep learning-based biomedical image segmentation. Nat Methods 18:203–211. 10.1038/s41592-020-01008-z 33288961 10.1038/s41592-020-01008-z

[14] Wasserthal J, Breit H‑C, Meyer MT et al (2023) TotalSegmentator: robust segmentation of 104 anatomic structures in CT images. Radiology 5:e230024. 10.1148/ryai.230024 37795137 10.1148/ryai.230024 PMC10546353

[15] Sotiras A, Davatzikos C, Paragios N (2013) Deformable medical image registration: a survey. IEEE Trans Med Imaging 32:1153–1190. 10.1109/TMI.2013.2265603 23739795 10.1109/TMI.2013.2265603 PMC3745275

[16] Viergever MA, Maintz JBA, Klein S et al (2016) A survey of medical image registration—under review. Med Image Anal 33:140–144. 10.1016/j.media.2016.06.030 27427472 10.1016/j.media.2016.06.030

[17] Fu Y, Lei Y, Wang T et al (2020) Deep learning in medical image registration: a review. Phys Med Biol 65:20TR01. 10.1088/1361-6560/ab843e 32217829 10.1088/1361-6560/ab843e PMC7759388

[18] Chen J, Liu Y, Wei S et al (2025) A survey on deep learning in medical image registration: new technologies, uncertainty, evaluation metrics, and beyond. Med Image Anal 100:103385. 10.1016/j.media.2024.103385 39612808 10.1016/j.media.2024.103385 PMC11730935

[19] Ehrhardt J, Lorenz C (2013) 4D modeling and estimation of respiratory motion for radiation therapy. 10.1007/978-3-642-36441-9

[20] Ehrhardt J, Werner R, Schmidt-Richberg A, Handels H (2011) Statistical modeling of 4D respiratory lung motion using diffeomorphic image registration. IEEE Trans Med Imaging 30:251–265. 10.1109/TMI.2010.2076299 20876013 10.1109/TMI.2010.2076299

[21] Huizinga W, Poot DHJ, Vernooij MW et al (2018) A spatio-temporal reference model of the aging brain. Neuroimage 169:11–22. 10.1016/j.neuroimage.2017.10.040 29203452 10.1016/j.neuroimage.2017.10.040

[22] Rohé M‑M, Datar M, Heimann T et al (2017) SVF-net: learning deformable image registration using shape matching. In: Descoteaux M, Maier-Hein L, Franz A et al (Hrsg) Medical Image Computing and Computer Assisted Intervention—MICCAI 2017. Springer, Cham, S 266–274

[23] Yang X, Kwitt R, Styner M, Niethammer M (2017) Quicksilver: fast predictive image registration—A deep learning approach. Neuroimage 158:378–396. 10.1016/j.neuroimage.2017.07.008 28705497 10.1016/j.neuroimage.2017.07.008 PMC6036629

[24] Miao S, Wang ZJ, Liao R (2016) A CNN regression approach for real-time 2D/3D registration. IEEE Trans Med Imaging 35:1352–1363. 10.1109/TMI.2016.2521800 26829785 10.1109/TMI.2016.2521800

[25] Uzunova H, Wilms M, Handels H, Ehrhardt J (2017) Training CNNs for image registration from few samples with model-based data augmentation. In: Descoteaux M, Maier-Hein L, Franz A et al (Hrsg) Medical Image Computing and Computer Assisted Intervention—MICCAI 2017. Springer, Cham, S 223–231

[26] Jaderberg M, Simonyan K, Zisserman A, Kavukcuoglu K (2015) Spatial transformer networks. Advances in Neural Information Processing Systems 28

[27] Li H, Fan Y (2018) Non-rigid image registration using self-supervised fully convolutional networks without training data, S 1075–1078 10.1109/ISBI.2018.8363757 PMC6070305 30079127

[28] Balakrishnan G, Zhao A, Sabuncu MR et al (2019) VoxelMorph: a learning framework for deformable medical image registration. IEEE Trans Med Imaging 38:1788–1800. 10.1109/TMI.2019.2897538 10.1109/TMI.2019.2897538 30716034

[29] Mok TCW, Chung ACS (2020) Large deformation Diffeomorphic image registration with Laplacian pyramid networks. Lecture Notes in Computer Science (including subseries Lecture Notes in Artificial Intelligence and Lecture Notes in Bioinformatics) 12263 LNCS, S 211–221 10.1007/978-3-030-59716-0_21

[30] Chen J, Frey EC, He Y et al (2022) TransMorph: transformer for unsupervised medical image registration. Med Image Anal 82:102615. 10.1016/j.media.2022.102615 36156420 10.1016/j.media.2022.102615 PMC9999483

[31] Hoffmann M, Billot B, Greve DN et al (2022) SynthMorph: learning contrast-invariant registration without acquired images. IEEE Trans Med Imaging 41:543–558. 10.1109/TMI.2021.3116879 34587005 10.1109/TMI.2021.3116879 PMC8891043

[32] Uzunova H, Wilms M, Forkert ND et al (2022) A systematic comparison of generative models for medical images. Int J CARS 17:1213–1224. 10.1007/s11548-022-02567-6 10.1007/s11548-022-02567-6 PMC9206635 35128605

[33] Lagogiannis I, Meissen F, Kaissis G, Rueckert D (2024) Unsupervised pathology detection: a deep dive into the state of the art. IEEE Trans Med Imaging 43:241–252. 10.1109/TMI.2023.3298093 37506004 10.1109/TMI.2023.3298093

[34] Uzunova H, Schultz S, Handels H, Ehrhardt J (2019) Unsupervised pathology detection in medical images using conditional variational autoencoders. Int J CARS 14:451–461. 10.1007/s11548-018-1898-0 10.1007/s11548-018-1898-0 30542975

[35] Kazerouni A, Aghdam EK, Heidari M et al (2023) Diffusion models in medical imaging: a comprehensive survey. Med Image Anal 88:102846. 10.1016/j.media.2023.102846 37295311 10.1016/j.media.2023.102846

[36] Uzunova H, Ehrhardt J, Handels H (2020) Generation of annotated brain tumor MRis with tumor-induced tissue deformations for training and assessment of neural networks. In: MICCAI 2020: Medical Image Computing and Computer Assisted Intervention—MICCAI 2020. Springer, Cham, S 501–511

[37] Yi X, Walia E, Babyn P (2019) Generative adversarial network in medical imaging: a review. Med Image Anal 58:101552. 10.1016/j.media.2019.101552 31521965 10.1016/j.media.2019.101552

[38] Eisemann N, Bunk S, Mukama T et al (2025) Nationwide real-world implementation of AI for cancer detection in population-based mammography screening. Nat Med. 10.1038/s41591-024-03408-6 39775040 10.1038/s41591-024-03408-6 PMC11922743

[39] Gulshan V, Peng L, Coram M et al (2016) Development and validation of a deep learning algorithm for detection of diabetic retinopathy in retinal fundus photographs. JAMA 316:2402. 10.1001/jama.2016.17216 27898976 10.1001/jama.2016.17216

[40] Schlegl T, Waldstein SM, Bogunovic H et al (2018) Fully automated detection and quantification of macular fluid in OCT using deep learning. Ophthalmology 125:549–558. 10.1016/j.ophtha.2017.10.031 29224926 10.1016/j.ophtha.2017.10.031

[41] Bogunovic H, Waldstein SM, Schlegl T et al (2017) Prediction of anti-VEGF treatment requirements in neovascular AMD using a machine learning approach. Invest Ophthalmol Vis Sci 58:3240. 10.1167/iovs.16-21053 28660277 10.1167/iovs.16-21053

[42] De Fauw J, Ledsam JR, Romera-Paredes B et al (2018) Clinically applicable deep learning for diagnosis and referral in retinal disease. Nat Med 24:1342–1350. 10.1038/s41591-018-0107-6 30104768 10.1038/s41591-018-0107-6

[43] Chopra R, Wagner SK, Keane PA (2021) Optical coherence tomography in the 2020s—outside the eye clinic. Eye 35:236–243. 10.1038/s41433-020-01263-6 33168975 10.1038/s41433-020-01263-6 PMC7853067

[44] Kepp T, Sudkamp H, von der Burchard C et al (2020) Segmentation of retinal low-cost optical coherence tomography images using deep learning. In: Medical Imaging 2020: Computer-Aided Diagnosis. International Society for Optics and Photonics, S 113141O

[45] Esteva A, Kuprel B, Novoa RA et al (2017) Dermatologist-level classification of skin cancer with deep neural networks. Nature 542:115–118. 10.1038/nature21056 28117445 10.1038/nature21056 PMC8382232

[46] Haenssle HA, Winkler JK, Fink C et al (2021) Skin lesions of face and scalp—Classification by a market-approved convolutional neural network in comparison with 64 dermatologists. Eur J Cancer 144:192–199. 10.1016/j.ejca.2020.11.034 33370644 10.1016/j.ejca.2020.11.034

[47] Bocanegra-Becerra JE, Neves Ferreira JS, Simoni G et al (2024) Machine learning algorithms for neurosurgical preoperative planning: a scoping review. World Neurosurg 194:123465. 10.1016/j.wneu.2024.11.048 39577649 10.1016/j.wneu.2024.11.048

[48] Chen X, Liu X, Wang Y et al (2022) Development and validation of an artificial intelligence preoperative planning system for total hip Arthroplasty. Front Med 9:841202. 10.3389/fmed.2022.841202 10.3389/fmed.2022.841202 PMC8981237 35391886

[49] Vasey B, Lippert KAN, Khan DZ et al (2023) Intraoperative applications of artificial intelligence in robotic surgery: a scoping review of current development stages and levels of autonomy. Ann Surg 278:896–903. 10.1097/SLA.0000000000005700 36177855 10.1097/SLA.0000000000005700 PMC10631501

[50] Guerrero DT, Asaad M, Rajesh A et al (2023) Advancing surgical education: the use of artificial intelligence in surgical training. Am Surg 89:49–54. 10.1177/00031348221101503 35570822 10.1177/00031348221101503

[51] https://www.med.upenn.edu/cbica/brats2020/data.html. Zugegriffen: 3. Febr. 2025

